# The Association of Autoimmune Disease With Lung Cancer Survival

**DOI:** 10.1001/jamanetworkopen.2020.30506

**Published:** 2020-12-01

**Authors:** Elizabeth R. Volkmann

**Affiliations:** Department of Medicine, David Geffen School of Medicine, University of California, Los Angeles.

A number of studies have demonstrated that patients with autoimmune and chronic inflammatory diseases are at heightened risk of the development of cancer, possibly due to factors such as aberrant immune function and surveillance,^1^ focal organ damage,^2^ and the use of immunomodulatory therapy.^3^ For example, 1 large study^4^ found that 13.5% of patients with lung cancer had an autoimmune disease diagnosed at any time before or after their cancer diagnosis. Despite the strong evidence associating autoimmune disease with lung cancer in particular, it is unclear how the presence of autoimmune disease affects morbidity and mortality in these patients. While some studies suggest that the presence of autoimmune disease adversely affects cancer-related outcomes,^5^ other studies have not substantiated these observations.^6^ In the current issue of *JAMA Network Open*, Jacob and colleagues^7^ compared lung cancer survival in patients with and without autoimmune disease and found no difference in survival between these 2 groups. Using a retrospective design of patients observed at a single academic center from 2003 to 2019, the authors identified 177 patients with biopsy-proven lung cancer and concomitant autoimmune disease and 219 patients with biopsy-proven lung cancer without autoimmune disease. Most patients were followed up for as long as 5 years after their cancer diagnosis. Only 14 and 19 patients in the autoimmune disease and control cohorts, respectively, were lost to follow-up during this time.

While the baseline demographic features, histopathology, and lung cancer stage of the 2 cohorts were similar (both were comprised predominantly of women, White individuals, and individuals with smoking history who had adenocarcinoma and locoregional stage at the time of diagnosis), the cancer treatment regimens varied. Fewer patients in the autoimmune disease cohort received standard-of-care initial treatment (126 [69.5%] vs 213 [97.3%]) as well as immunotherapy (8 [4.5%] vs 74 [33.8%]) compared with the control cohort. Among the autoimmune disease cohort, the most common reason for not receiving standard-of-care cancer treatment was poor performance status and/or frailty. Despite the observed treatment regimen disparities, there was no difference in progression-free survival by stage between the 2 cohorts. Moreover, in a multivariable Cox proportional hazards model analysis, the only independent factor associated with worse progression-free survival was increased age, while sex, race, smoking status, and the presence of an autoimmune disease were not significantly associated. Similar results were found for the analysis of overall survival and recurrence in locoregional disease. Taken together, the results of this study suggest that the presence of an autoimmune disease does not adversely affect cancer-related outcomes, at least during a 5-year follow up period.

In a subgroup analysis, the authors also found no difference in survival among patients with specific autoimmune diseases (eg, rheumatoid arthritis, systemic lupus erythematosus, systemic sclerosis) or based on the presence of specific comorbidities (eg, interstitial lung disease [ILD]). However, these analyses may have been underpowered to detect significant differences.

A notable strength of the present study design included the comprehensive characterization of the 2 cohorts through an extensive medical record review. Although the use of large databases in oncology outcomes research affords the study of larger sample sizes and more diverse patient populations, these databases often possess variations in coding, unrecorded variables, and selection bias as well as incomplete assessment of clinically relevant end points. Thus, although the sample size of the present study was relatively small, the small number of patients lost to follow-up provides reassuring evidence that the present findings are unlikely to be due to chance alone.

However, it is unclear whether the results are generalizable to other patient populations, namely patients who receive care outside of an academic medical center. The authors astutely noted that survival in both groups was better than expected relative to the general population. It is also conceivable that both lead time bias and length bias contributed to improved survival in patients with autoimmune disease. For instance, patients at risk of autoimmune-associated ILD often undergo screening for ILD with high-resolution computed tomography imaging. Length time bias is a particular concern in this scenario because certain detected cancers may be slowly growing and may have otherwise never come to clinical attention in the patient’s lifetime had screening not occurred.

It is also unclear how the differences in treatment strategies between the 2 cohorts may have contributed to the observed outcomes. Substantially more patients in the control cohort received immunotherapy for their cancer, and the reasons for this difference are not entirely clear. While the preexistence of autoimmune conditions may predispose patients to more severe complications from such therapy, this is a theoretical concern and warrants further investigation.

In summary, the present study provides compelling evidence that the existence of an autoimmune condition does not adversely affect survival in patients with lung cancer. Future studies are needed to evaluate whether specific immunomodulatory agents used to treat autoimmune conditions as well as the duration of immunomodulatory therapy affect lung cancer survival rates in these patients. The answers to these questions may help guide future therapeutic efforts in this area.
